# Improving expression and assembly of difficult-to-express heterologous proteins in *Saccharomyces cerevisiae* by culturing at a sub-physiological temperature

**DOI:** 10.1186/s12934-023-02065-7

**Published:** 2023-03-23

**Authors:** Kum-Kang So, Ngoc My Tieu Le, Ngoc-Luong Nguyen, Dae-Hyuk Kim

**Affiliations:** 1 Institute for Molecular Biology and Genetics, Department of Molecular Biology, Jeonbuk National University, Jeonju, Jeollabuk-Do 54896 Republic of Korea; 2 Department of Bioactive Material Sciences, Jeonbuk National University, Jeonju, Jeollabuk-Do 54896 Republic of Korea; 3 Department of Biology, College of Sciences, Hue University, Hue, 530000 Vietnam

**Keywords:** *Saccharomyces cerevisiae*, Expression temperature, LTB-fusion protein

## Abstract

**Background:**

*Escherichia coli* heat labile toxin B subunit (LTB) is one of the most popular oral vaccine adjuvants and intestine adsorption enhancers. It is often expressed as a fusion partner with target antigens to enhance their immunogenicity as well as gut absorbability. However, high expression levels of a fusion protein are critical to the outcome of immunization experiments and the success of subsequent vaccine development efforts. In order to improve the expression and functional assembly of LTB-fusion proteins using *Saccharomyces cerevisiae*, we compared their expression under culture conditions at a sub-physiological temperature 20 °C with their expression under a standard 30 °C.

**Results:**

The assembled expression of LTB-EDIII_2_ (LTB fused to the envelope domain III (EDIII) of Dengue virus serotype 2), which was expressed at the level of 20 µg/L in our previous study, was higher when the expression temperature was 20 °C as opposed to 30 °C. We also tested whether the expression and functional assembly of a difficult-to-express LTB fusion protein could be increased. The assembled expression of the difficult-to-express LTB-VP1 fusion protein (LTB fused to VP1 antigen of Foot-and-Mouth Disease Virus) dramatically increased, although the total amount of expressed protein was still lower than that of LTB-EDIII_2_. Slight but significant increase in the expression of well-known reporter protein eGFP, which has previously been shown to be increased by cultivation at 20 °C, was also observed in our expression system. As no significant changes in corresponding transcripts levels and cell growth were observed between 20 °C and 30 °C, we infer that translation and post-translational assembly are responsible for these enhancements.

**Conclusions:**

The effects of lowering the expression temperature from 30 °C to 20 °C on protein expression and folding levels in *S. cerevisiae*, using several proteins as models, are reported. When heterologous proteins are expressed at 20 °C, a greater amount of (specially, more assembled) functional proteins accumulated than at 30 °C. Although further studies are required to understand the molecular mechanisms, our results suggest that lowering the expression temperature is a convenient strategy for improving the expression of relatively complexly structured and difficult-to-express proteins in *S. cerevisiae*.

**Supplementary Information:**

The online version contains supplementary material available at 10.1186/s12934-023-02065-7.

## Background

Mucosal vaccines, which are typically delivered through nasal or oral routes, offer an attractive solution to some of the challenges of mass vaccination programs due to their cost-effectiveness, safety, and high rate of public acceptance [1, 2]. Despite these benefits, far fewer mucosal than injectable vaccines have been approved. One significant obstacle to mucosal vaccine development is their tendency to induce low immunogenicity, sometimes even promoting the target antigens to develop tolerance. This is the result of difficulties in controlling the quantity of antigens delivered to the immune system through the mucosal interfaces. Thus, specifically in the context of mucosal vaccines, the choice of adjuvants plays a much more crucial role in determining efficacy and protective effect [2]. Among the most potent and widely used mucosal adjuvants are the *Vibrio cholera* and *Escherichia coli* toxins and their mutant forms (dmLT and mmCT, respectively) [2–5]. These adjuvants are unique in that they consistently induce a very strong mucosal immune response specific to the target antigens as well as a systematic response when delivered orally or intranasally [5].

Baker’s yeast, *Saccharomyces cerevisiae*, is the most popular eukaryotic host for the expression of foreign proteins [6, 7], because as a single cell microorganism, *S. cerevisiae* combines the advantages of simple prokaryotic systems—including high expression level, ease of genetic manipulation and scale-up, fully developed mass culturing—with those of eukaryotic post-translational modifications and secretion. Moreover, *S. cerevisiae* is a generally recognized as safe (GRAS) organism, to make its expressed product and processing more applicable without further consideration. In addition, its high-quality protein content and high vitamin levels distinguish *S. cerevisiae* as a favorable single cell organism useful for live and oral administration of pharmaceutical and feed products [8, 9]. Furthermore, the strong adjuvant properties of yeast derivatives render it an attractive heterologous expression system for vaccine production and development [10–17]. A well-balanced immune response to dengue was observed when a yeast expression system was used to produce dengue virus epitope proteins [18].

One common oral vaccine development strategy is to express target antigens intracellularly fused with the *E. coli* heat labile toxin B subunit (LTB) in the yeast [13, 16]. This fusion protein is capable of inducing strong systematic as well as mucosal immune responses against the target antigens. However, if the availability of the antigen is limited, such as in a vaccine in which the whole yeast cell is used instead of the protein extract, the strength of the immune responses is compromised [10]. To overcome this problem, it is suggested improving the expression levels of the target antigen. This goal could be achieved through systematic development of various recombinant technologies including codon optimization [19], strong promoters and terminators [20–22], and multi-copy expression vectors [23]. In addition, some have suggested altering cultural practices such as acting to change the expression temperature from the physiological temperature [24, 25].

Our previous studies, which were focused on the development of an oral vaccine, used *S. cerevisiae* to express LTB fused with a Dengue antigen [13] that induced immune responses in mice. We found that oral vaccine efficacy might be further improved by increasing LTB-fusion protein expression. Lowering expression temperatures is an established and popular approach to improve the production of functional heterologous proteins in *Escherichia coli* [26], as heterologous proteins tend to aggregate at high temperatures [27] but the induction temperature does not seem to improve overall expression level. In animal cells such as CHO, lowering expression temperature also has a positive effect on target protein expression [28–30]. Although there is a general consensus, supported by some reports, concerning improved heterologous expression in *P. pastoris* [31–33], *Yarrrowia lipolitica* [34], *Kluyveromyces lactis* [35, 36] and *S. cerevisiae* [24] by temperature reduction, these lacked a systematic approach that would show that this is a widespread phenomenon with practical applicability. As we explored potential means of improving antigen expression using *S. cerevisiae*, we observed that expression levels and the functional assembly of target proteins increased significantly when expression temperature was lowered from 30 °C to 20 °C. In an attempt to determine the underlying mechanisms, we examined the effect of lowering the expression temperature on three different constructs which collectively covered a wide range of expression levels. We tested our expression system for eGFP as a model reporter protein with a strong expression level [24] in *S. cerevisiae* whose temperature was lowered to 20 °C. We also tested our previous LTB-EDIII_2_ fusion protein (LTB fused to envelope domain III (EDIII) of Dengue virus serotype 2), which has a lower expression level (20 g/L) than LTB-scEDIII (LTB fused to the synthetic consensus sequence of EDIII of all four serotypes of Dengue virus) (4 mg/L), as a low expression target [13], and LTB-VP1 (LTB fused to VP1 antigen Foot-and-Mouth Disease Virus) as a difficult-to-express target. Our results revealed that the benefits of lowering expression temperature do not stop at protein assembly but extend to total expression level. This report also highlights a convenient strategy for the modulation of heterologous protein expression in *S. cerevisiae*, as well as the need to explore the underlying mechanisms so that its potential can be fully exploited.

## Results

### Expression analyses of eGFP

**Fig. 1:**
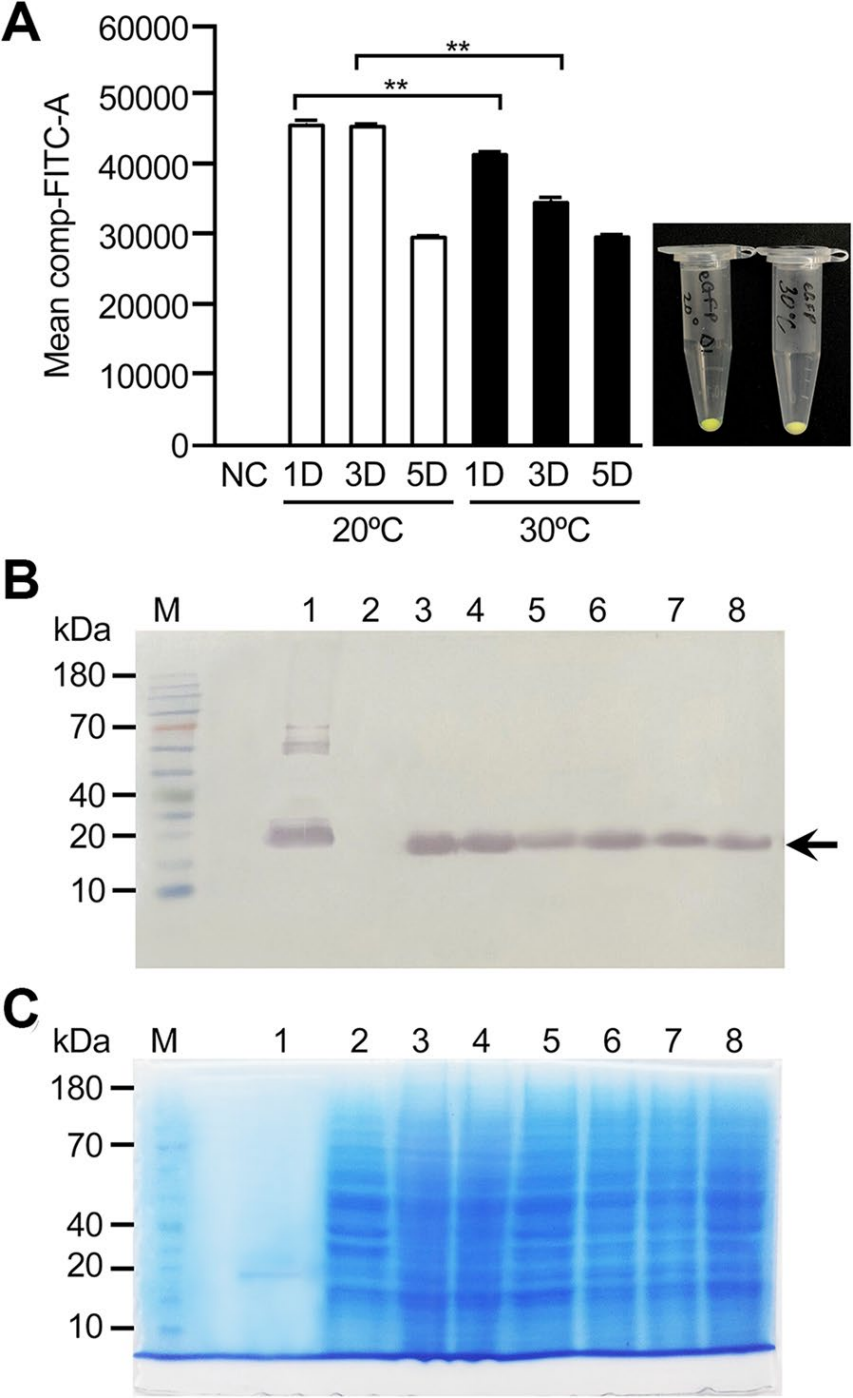
Expression analysis of yeast codon optimized eGFP under two different expression conditions using FACS (**A**) and Western blot analysis (**B** and **C**). **A** FACS analysis of expressed eGFP. Inset: yeast cells cultured at 20 °C (left) and 30 °C condition (right) show eGFP expression under UV light. **B** Western blot of yeast expressing eGFP in 20 °C and 30 °C conditions. Lane 1: purified eGFP; lane 2: *S. cerevisiae* 2805 strain; lanes 3–5: protein preparation from eGFP-expressing yeast cells harvested after 1, 3, and 5 days, respectively, at 20 °C. Lanes 6–8: protein preparation from eGFP-expressing yeast cells harvested after 1, 3, and 5 days, respectively, at 30 °C. **C** SDS-PAGE twin gel showing that a similar amount of protein was loaded on each lane

Since FACS analysis can be easily applied to measure cell fluorescence without further treatment, the expression levels of reporter protein eGFP were compared among transformants (Fig. 1A) by FACS analysis. Twenty transformants were selected from the ura^−^ selective media and then analyzed by FACS. There was little variation in levels of eGFP expression among tested transformants. Among the examined transformants, we selected one for further analysis by FACS and Western blot analysis of the temporal expression of eGFP at 20 °C and 30 °C. As seen in Fig. 1A, the FACS showed that eGFP expression in the sample cultivated at 20 °C was slightly but noticeably stronger on days 1 and 3 than in those cultivated at 30 °C. The expression of eGFP in this culture eventually decreased, and ultimately no significant difference between the 20 °C and 30 °C cultures was observed 5 days after cultivation. Western blot results were consistent with those of the FACS analysis (Fig. 1B).

### Expression analyses of LTB-EDIII_2_

**Fig. 2:**
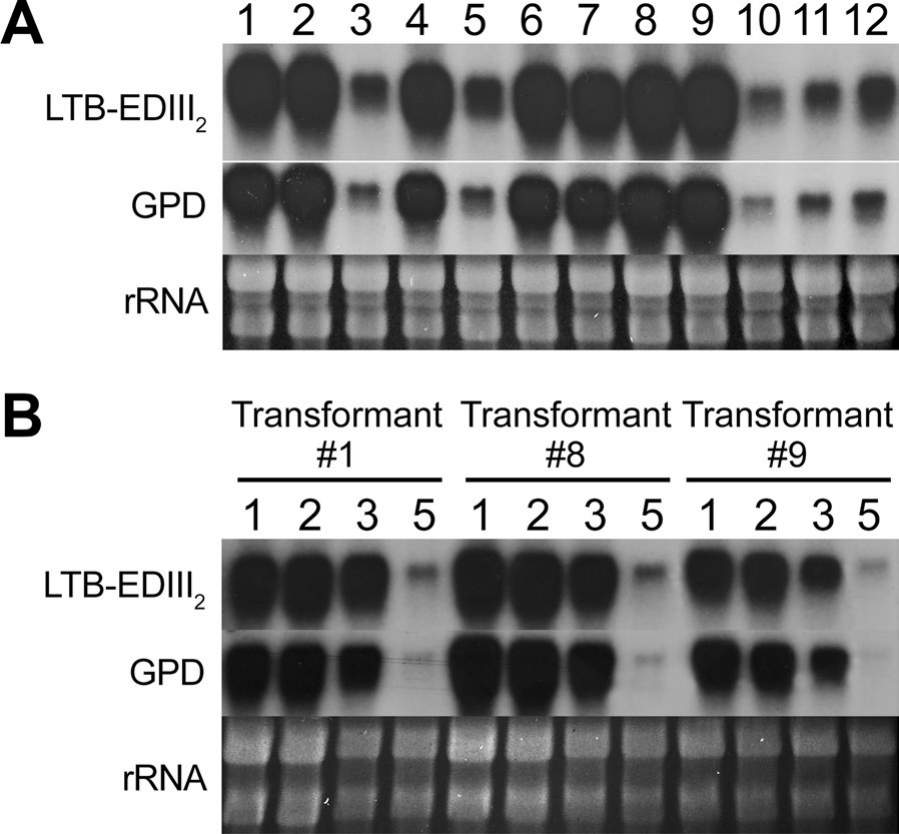
**A** Northern blot analysis of 12 transformants. 30 µg of total RNA was loaded on each lane. LTB-EDIII_2_ was used as the probe to detect LTB-EDIII_2_ expression. Lanes 1–12: LTB-EDIII_2_ transformants were randomly selected and numbered from 1 to 12. **B** Temporal expression of LTB-EDIII_2_ transcript in three selected transformants (transformants #1, #8, and #9 in panel **A**). Lane numbers indicate the number of days after cultivation at 30 °C and strains are indicated at the top of the lanes. Glyceraldehyde-3-phosphate dehydrogenase gene (GPD) was used as an internal control and bands representing rRNAs in ethidium bromide strained-gel (rRNA) are shown to indicate an equal amount of RNA loaded on each lane

We tested LTB-EDIII_2_ as an exemplar “low expression” LTB-fusion protein. We choose LTB-EDIII_2_ as its expression was significantly lower than that of a similar LTB-scEDIII fusion protein; approximately, 200 times less LTB-EDIII_2_ was expressed than LTB-scEDIII (20 g/L vs. 4.0 mg/L). Northern blot and Western blot screenings of all candidate transformants showed that the target proteins were indeed expressed in all of the transformants. From the initial Northern blot (Fig. 2A) screening of LTB-EDIII_2_ expression, three colonies (#1, #8 and #9) showing intensive hybridizing bands were selected for further temporal expression analysis at the transcription level. At 30 °C, the expression patterns of the three-selected LTB-EDIII_2_ transformants peaked to 1—3 days after cultivation (Fig. 2B), a result that is consistent with our previous studies [13].

**Fig. 3:**
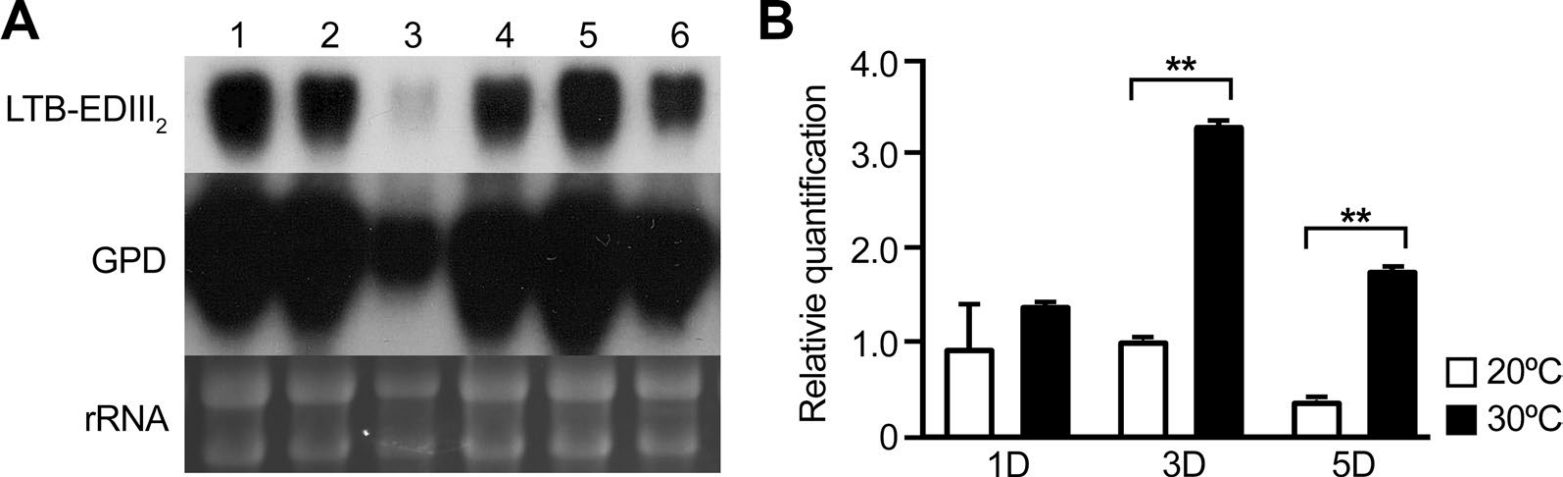
Northern blot analysis (**A**) and Quantitative real time RT-PCR (qRT-PCR) analysis (**B**) of LTB-EDIII_2_ in a selected transformant (#8) under 20 °C and 30 °C conditions. **A** 20 µg total RNA was loaded on each lane. RNA preparations from cells harvested at day 1, 3, and 5 days after cultivation at 20 °C (Lanes 1–3, respectively) and 30 °C (Lanes 4–6, respectively). GPD was used as an internal control and rRNAs are shown to indicate equal amount of RNA loaded on each lane. **B** qRT-PCR results of changes in expression of LTB-EDIII_2_ under 20 °C and 30 °C conditions are shown. Error bars indicate standard deviation based on three independent measurements. ** indicates statistically significant difference between two groups, according to *t*-test at *p* = 0.01

We then selected transformant #8 as the representative transformant used a Northern blot analysis to compare the temporal expression patterns of LTB-EDIII_2_ at 20 °C and 30 °C. As shown Fig. 3A, no considerable difference in transcript accumulation was observed until 3 days after cultivation at either 20–30 °C. Although a dramatic decrease of in the band intensity of the LTB-EDIII_2_ transcript was observed at 5 days after cultivation, it was less so once it was normalized to the internal control gene (GPD) expression. Only a slight but significant decrease in band intensity was estimated after the normalization. We then checked the quantitative gene expression levels using real-time RT PCR, and the results indicated that transcript accumulation at 30 °C was stronger than that at 20 °C over the surveyed period. This suggests that transcription of the LTB-EDIII_2_ gene may not be a major factor in the production of the target protein and, if any, 30 °C is a more favorable condition for transcription than 20 °C (Fig. 3B).

**Fig. 4:**
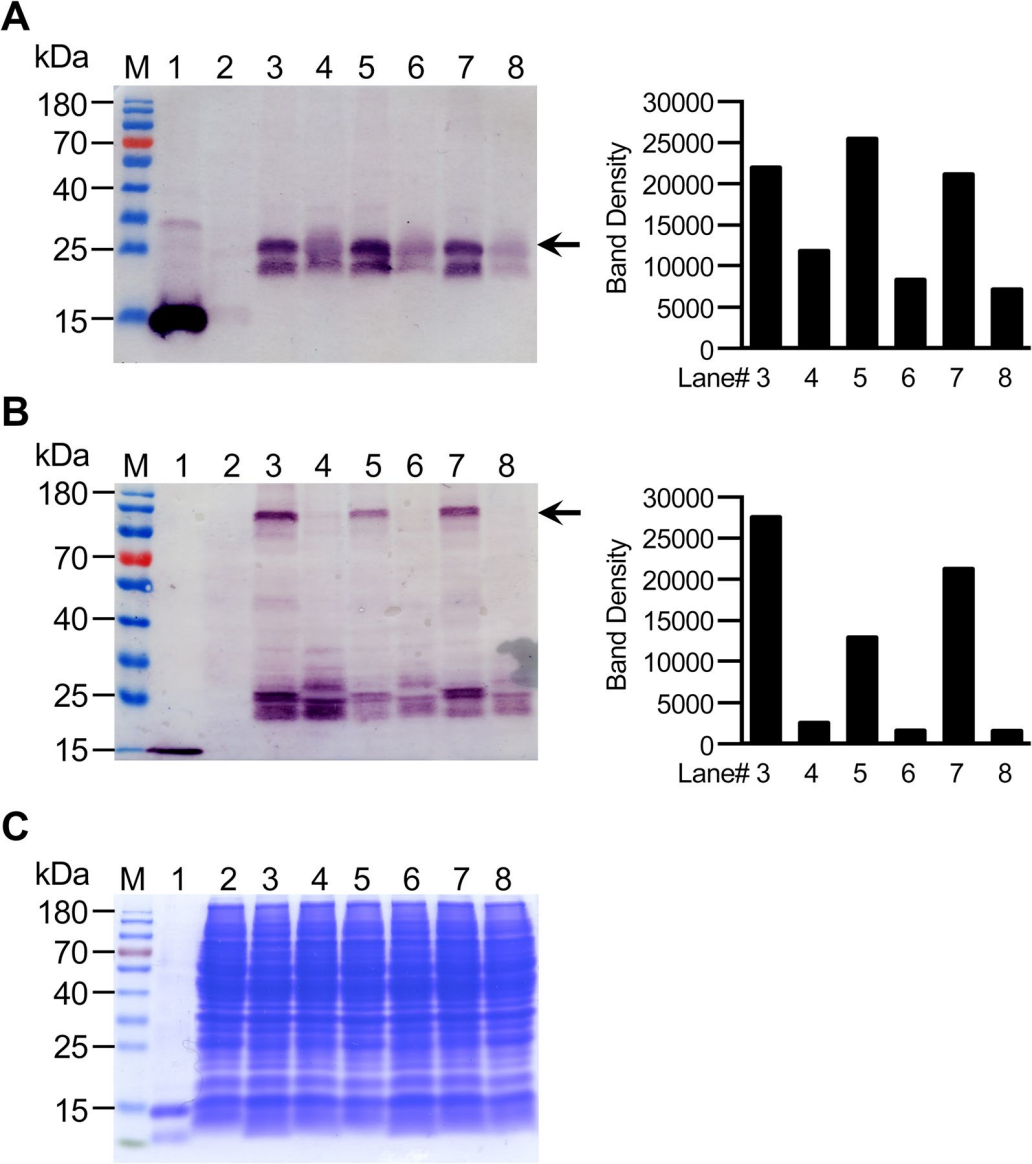
Western blot analysis of three selected LTB-EDIII_2_ transformants. **A** LTB-EDIII_2_ detection under denatured condition. **B** LTB-EDIII_2_ detection under non-denatured condition. Densitometry analysis of cross-reacting LTB-EDIII_2_ bands in the corresponding blot is shown on the right panel. **C** SDS-PAGE gel shows that an equal amount of protein was loaded on each lane. Lane 1: purified *E. coli*-expressed EDIII_2_ as a positive control; Lane 2: a mock transformant as a negative control; Lanes 3, 5 and 7: LTB-EDIII_2_ transformants #1, #8, and #9 cultured at 20 °C; Lanes 4, 6 and 8: LTB-EDIII_2_ transformants #1, #8, and #9 cultured at 30 °C. Proteins were prepared from cells cultured for 3 days after the inoculation to the expression medium

**Fig. 5:**
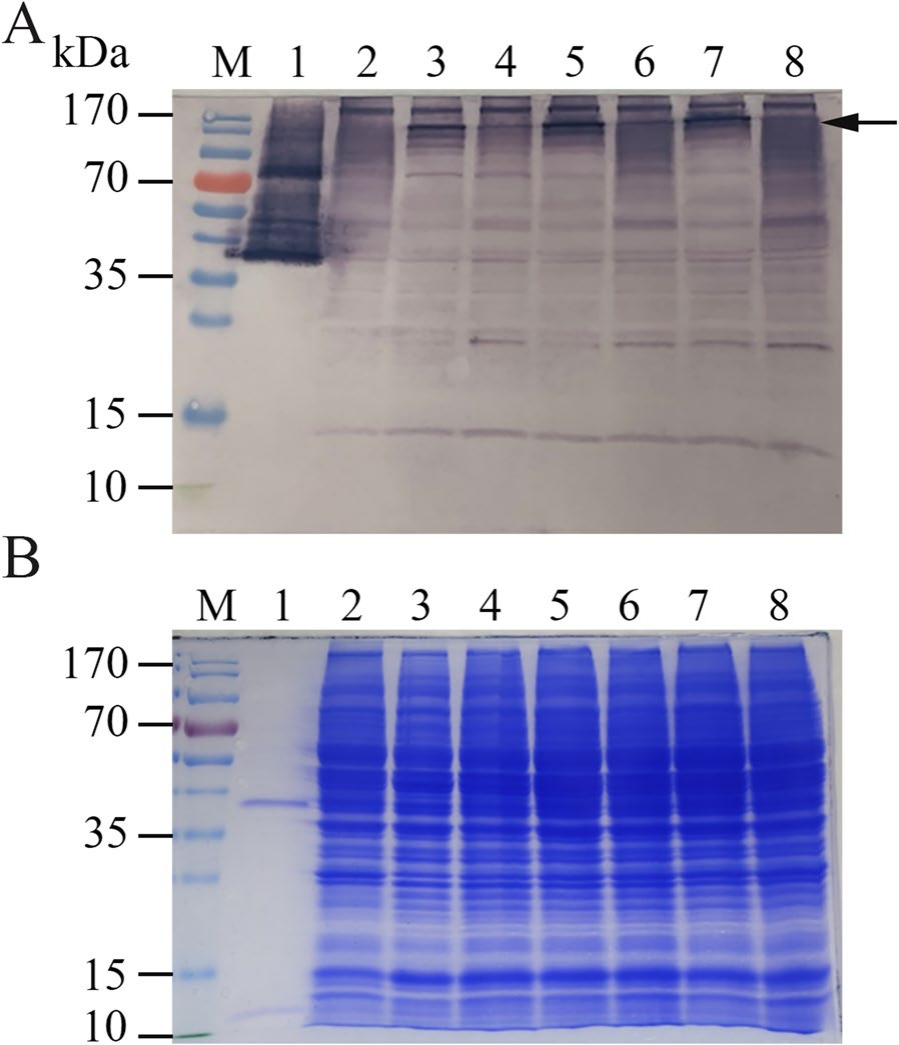
Western blot analysis of temporal expression of LTB-EDIII_2_ from transformant #8. **A** LTB-EDIII_2_ expression was resolved under non-denaturing condition at 20 °C and 30 °C. **B** SDS-PAGE gel showed that an equal amount of protein was loaded on each lane. Lane 1: purified *E. coli*-expressed LTB as a positive control; Lane 2: a mock transformant cultured for 3 days as a negative control. Proteins were prepared from transformant #8, and cultured for 1, 3, 5 days after inoculation to the expression medium at 20 °C (lanes 3, 5, and 7, respectively) and 30 °C (lanes 4, 6, and 8, respectively)

To examine the production of corresponding LTB-EDIII_2_ protein, three selected transformants were subjected to Western blot analysis under both non-denaturing (non-boiling) and denaturing (boiling) conditions. As shown in Fig. 5, LTB-EDIII_2_ protein expression after 3 days was dramatically different at 20 °C and 30 °C, i.e., the LTB-EDIII_2_ protein from all three strains was expressed much higher levels at 20 °C than at 30 °C (Fig. 4A). Band intensity was twice or three times as high at 20 °C than at 30 °C (Fig. 4A). Interestingly, the difference in the level of expression between 20 °C and 30 °C under the denatured condition (Fig. 4A), which designed to detect the monomeric LTB-EDIII_2_, was noticeably high, but still not as high as the difference observed in the non-denatured condition (Fig. 4B), which we tested to detect the assembled oligomeric forms of LTB fusion protein. At least 8 times more assembled LTB-EDIII_2_ was detected at 20 °C than at 30 °C (Fig. 4B). These results suggest that cultivation at 20 °C not only increased LTB-EDIII_2_ monomer production but also greatly facilitated the assembly of the expressed monomeric subunit.

**Fig. 6:**
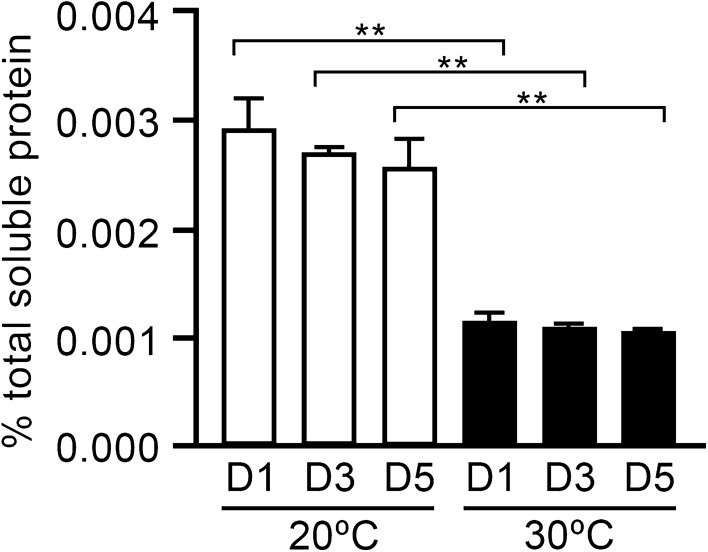
GM1-ganglioside binding assay (GM1 ELISA) of LTB-EDIII_2_. The ELISA was conducted by coating 96-wells with GM1 monosialoganglioside as the receptor molecule. Y-axis indicates the percentage of pentameric LTB-EDIII_2_ in the sample preparations. The coated plates were incubated with the protein preparation of transformant #8 and then cross-reacted with anti-LTB antiserum

Temporal expression of LTB-EDIII_2_ in transformant #8 was performed at days 1, 3 and 5 using Western blot analysis (Fig. 5, Additional file 1: Fig. S1) and GM1 ELISA (Fig. 6). The Western analysis indicated that differences in LTB-EDIII_2_ expression were hardly dependent on culture period. However, significantly more assembled LTB-EDIII_2_ was produced at 20 °C than at 30 °C (Fig. 5). GM1 ELISA, which quantitatively measures the amount of pentameric LTB-EDIII_2_, indicated that the yield of the assembled pentameric form of LTB-EDIII_2_ was 2.5 greater at 20 °C than at 30 °C (Fig. 6). As no significant changes to transcript levels were observed between the 20 °C and 30 °C tests, these results suggest that the improved expression of the target proteins is not be due to transcriptional factors, but rather translational and/or post-translational levels.

### Expression analyses of LTB-VP1

**Fig. 7:**
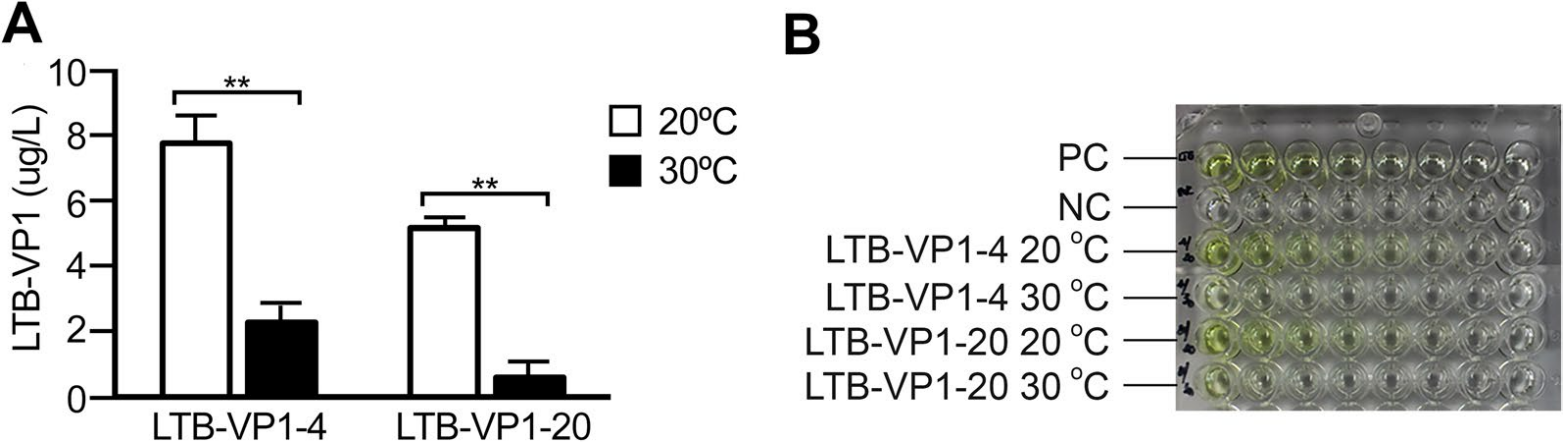
GM1-ganglioside binding assay (GM1 ELISA) of LTB-VP1. **A** ELISA was conducted as described earlier. The plates coated with GM1 monosialoganglioside were incubated with the protein preparation of two selected LTB-VP1 transformants and then cross-reacted with anti-LTB antiserum. **B** Plates are shown to demonstrate colorimetric changes in the well, in contrast with samples from the 30 °C cultures. PC and NC indicate that the *E. coli*-expressed LTB as a positive control and a mock transformant as a negative control, respectively. Well number 1 indicates the sample of protein preparation without dilution and well numbers (2—8) indicate samples with twofold dilution of the original protein preparation

As an example of a “difficult-to-express” LTB-fusion protein, we tested the expression of the LTB-VP1. In our attempt to express epitopes of Foot-and-Mouth Disease Virus (FMDV), we found that LTB-VP1 as well as VP1 (comprising aa 725—935 of FMDV (GenBank accession No: AY593823.1)) was difficult-to-express, whereas the remainder of the viral structural proteins (e.g., VP0, VP2, VP3, and VP4) were relatively easier to express (data not shown). We then tested whether the LTB fusion protein (LTB-VP1) could be quantified using GM1 ELISA. The LTB-VP1 transformants were also subjected to a procedure similar to those described above applicable to LTB-EDIII_2_. Three transformants were selected after a Northern blot analysis, after which one among the three was subjected to temporal expression analysis by additional Northern blot and real-time RT PCR (Additional file 2: Fig. S2). Protein expression was also analyzed by Western blot and GM1 ELISA. As predicted, Western blot analysis using anti-LTB antibody failed to detect discernable bands associated with any monomeric subunit (denatured condition) or multimeric assembly (non-denatured condition) of LTB-VP1 (data not shown). With this indication that levels of LTB-VP1 expression were so low as to be undetectable by Western blot analysis, we performed a more sensitive GM1-ELISA assay. As shown in Fig. 7, a very low GM1-ELISA signal was observed from samples cultured at 30 °C even at the lowest (1/16) dilutions. However, although not as strong as those observed from LTB-EDIII_2_, the signals indicating assembled LTB-VP1 were so distinctive in the samples cultured at 20 °C that a colorimetric outcome of reaction mixture rendered the change apparent to the naked eye. Based on GM1-ELISA, we estimated the amount of assembled pentameric forms of LTB-VP1 at 20 °C to be 7.8 g/L, which is at least 3 times more than the amount of assembled LTB-VP1.

## Discussion

*S. cerevisiae* has been one of the most popular heterologous expression hosts in biotechnology, and it is increasingly employed in oral vaccine development [10–16, 37–41]. Our previous study [13] showed that the expression of LTB fused with a synthetic Dengue tetravalent antigen using *S. cerevisiae* was potentially useful as an oral vaccine against Dengue viruses. However, the intensity of the immune response elicited seem to depend on the bioavailability of the target antigen, i.e. the amount of the target antigen available for absorption by gut mucosal lymphoid organs such as Peyer’s patches. In this paper, to increase the bioavailability of the antigen, we prepared and partially concentrated cell free extract instead of using the entire cell for vaccination. Unfortunately, this step requires extra preparation in terms of effort and equipment, runs the risk of contaminating the oral vaccines, and is therefore undesirable in the context of oral vaccine development. If instead whole yeast cells are used, the expression level of the target antigen must be increased. Although the production of recombinant protein in *S. cerevisiae* is straightforward, improving the yield of a heterologous protein using this fungus is a more complicated process. While various strategies for improving heterologous expression in *S. cerevisiae* have been described, such as the selection of strong promoters and enhancers [20, 21], changing terminators [22, 42, 43], engineering protein translocation by overexpressing protein folding enhancing genes [44], or codon optimization [19], each necessitates the extra effort of redesigning the expression systems and adapting the new systems to current laboratory conditions. As an alternative, in one intriguing work expression as well as secretion of GFP in *S. cerevisiae* was observed to improve by lowering the expression temperature to 20 °C [24]. It remained unclear, however, whether this phenomenon would be universally applicable to all heterologous proteins expressed in the yeast, or only some of them.

In this work, we surveyed three recombinant proteins expressed in the *S. cerevisiae* 2805 strain. We observed that the yields of all three proteins were increased by lowering the temperature to 20 °C from the yeast physiological temperature at 30 °C during the expression phase [45]. Most importantly, the amount of assembled multimeric LTB-fusion protein increased dramatically, and was particularly more pronounced in the proteins with a multimeric structure or otherwise low expression levels, (e.g., LTB-EDIII_2_ and LTB-VP1, respectively) and those that are less conspicuous in the protein but possess a relatively simple structure and expressed well (e.g., eGFP). Northern blot and real-time RT PCR of LTB-EDIII_2_ showed that the increase in the production of the target protein was not due to changes in transcription.

The effects of various types of stress on recombinant protein production in yeast were reviewed [46]. The stress factors that may affect the expression of recombinant proteins can be divided into two groups: metabolic stresses arising from interference with cellular processes and environmental stresses due to culture conditions. The most notable cellular response to metabolic stress is the unfolded protein response, which implicates the limited processing capacity of the endoplasmic reticulum (ER) as well as various quality control mechanisms in degrading unfolded, misfolded, and aggregated heterologous proteins. The use of multicopy expression plasmids may induce extra-metabolic stress on cells and may offset their transcriptional advantage, resulting in low expression levels. In terms of environmental stresses, reducing expression temperature may inhibit protease degradation, leading to an increase in the amount of heterologous proteins [46].

Reducing the expression temperature in *E. coli* has been widely exploited as a solution to the problem of too many inclusion bodies vs too little soluble expression [47–49]. Reduction of the post-induction temperature has been thought to reduce the translational rate, thereby enhancing solubility. High temperatures are also thought to be conducive to hydrophobic aggregation [27]. Some previous studies have also reported benefits associated with expressing heterologous proteins in *P. pastoris* at low temperatures and proposed that the improvement in the target proteins was due to the enhancement of the protein folding pathway and reduced cell death [50–52]. However, Hohenblum et al. [53] showed that cell viability was not affected by temperature. Similar observations have also been reported in animal cells [28–30, 54], suggesting that the phenomenon is a universal one. To date, however, attempts to understand the underlying mechanisms involving transcriptomic [55] and proteomic [56] surveys of *S. cerevisiae* have not yielded a satisfactory explanation for the observed phenomenon. A similar conclusion was also reached by Alexandra Graf [57].

**Fig. 8:**
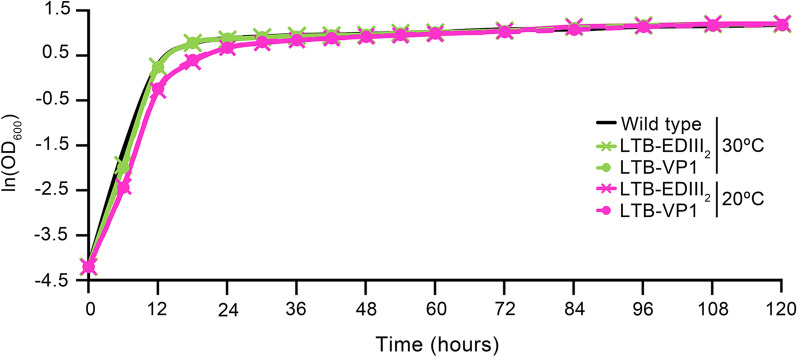
Growth curve of transformants. Cell growth was monitored by measuring optical density at 600 nm using a spectrophotometer (OD_600_). The growth curves are shown as representative profiles based on duplicated experiments with three replications

We observed that when *E. coli*, yeast, or animal cells are forced to express heterologous protein at sub-optimal temperatures, cells grew more slowly than they would have at the optimal temperatures. When the temperature is decreased further, such as to 15 °C or below 10 °C, cell mass decreases dramatically or cells may even stop dividing [55]. However, in the case of yeast, its growth rate at 20 °C is slightly, but not dramatically, lower (lnOD 0.36 at 18 h) from the early growth phase than at 30 °C (lnOD 0.76 at 18 h) (Fig. 8). Moreover, our cultivation procedure for yeast expression was designed to inoculate a large quantity of exponentially growing cells within the nutrient rich media, i.e. successively cultured cells (approximately 10^5^cells per mL) in a total volume of 40 mL YEPD media. This system was designed to support active cell growth and gene expression at different temperatures (20 °C or 30 °C). This inoculation strategy allowed the culture system to reach the stationary phase less than 36 h after incubation regardless of whether the culture was maintained at 20 °C or 30 °C (Fig. 8). Balancing between fermentation and respiration is essential to achieving adequate protein production levels [58, 59]. Accordingly, for the process tested in this paper, 20 °C seems to be the ideal temperature that maximizes protein expression without needlessly compromising cell growth. In addition, the stability of the introduced plasmids in the yeast was measured at the end of the cultivation period by comparing the number of colony forming units (CFUs) per ura^−^ selective and non-selective plate [16, 60]. The plasmid stability of all transformants allowed more than 82% of the plated cells to maintain the plasmids, and we observed no difference in stability between 20 °C and 30 °C samples. These results suggested that plasmid stability should be excluded as potential explanations for the difference in expression levels.

Based on the fact that more functional proteins were produced at the sub-optimal temperature, we hypothesize that protein folding plays an important part in the improved expression. Since GPD is a strong constitutive promoter, the target genes were likely overexpressed at the transcriptional level, which was an observation that is consistent with the results of our Northern blot analysis. This may result in the protein folding system becoming overwhelmed. Likewise, the overexpression of proteins involved in protein folding improves the yield of heterologous proteins [44, 61]. In short, although further studies are required for verification, it is likely that the increase in functional protein amounts observed in cultures at low temperatures is a consequence of both the translation machinery as well as the protein folding system.

## Conclusions

In this study we reported the effects of a lowered expression temperature (from 30 °C to 20 °C) on protein expression levels and protein folding levels in *S. cerevisiae*, using several proteins as models. When heterologous proteins were expressed at 20 °C, which is a sub-physiological temperature of *S. cerevisiae*, more proteins were produced and more assembled functional proteins were accumulated, than when these same proteins were expressed at 30 °C. Although further studies will be required before the molecular mechanisms implicated by this result is fully understood, our results suggest that cell integrity, plasmid stability, and enhanced protein folding play a role. To our knowledge, this is the first report to test this novel strategy for improving heterologous protein production in *S. cerevisiae*.

## Methods

### Strains and culture conditions

*Escherichia coli* Top10 was used throughout the various stages described herein for cloning. BL21 (DE3)-RIPL and BL21 (DE3) were employed to produce recombinant antigens, which were used as standards in Western and ELISA analyses. All *E. coli* strains were maintained in LB broth or on LB agar supplemented with appropriate antibiotics. *Saccharomyces cerevisiae* 2805 strain (*MATα pep4::HIS3 prb 1-δ Can1 GAL2 his3 ura3-52*) was used as a recipient to express all of the LTB fusion proteins mentioned in this report. *S. cerevisiae* 2805 was maintained in complete medium YEPD (Yeast Extract Peptone Dextrose) before transformation, and subsequently in Ura dropout medium (0.56% Yeast Nitrogen Base with ammonium sulfate, 0.76% KCl, 2% Glucose and 0.14% Yeast synthetic dropout, all in weight/volume unit; and supplemented with 20 mg/L of tryptophan, histidine, leucine and adenine hemisulfate).

Both *E. coli* and *S. cerevisiae* were transformed by chemical methods described previously [18]. For yeast expression, a single colony was inoculated into 5 ml of Ura dropout liquid medium and cultured for 48 h at 200 rpm and 30 °C in a shaking incubator. Subsequently, 250 µL of this culture was transferred into 5 ml YEPD medium and cultured for another 16 h under these same conditions. This culture was then inoculated into 40 ml of freshly prepared YEPD medium and grown at either 30 °C or 20 °C at 200 rpm. Biomasses were collected at days 1, 3 and 5 post-inoculation, weighted and stored at − 80 °C for expression analyses.

### Plasmid constructions

**Fig. 9:**
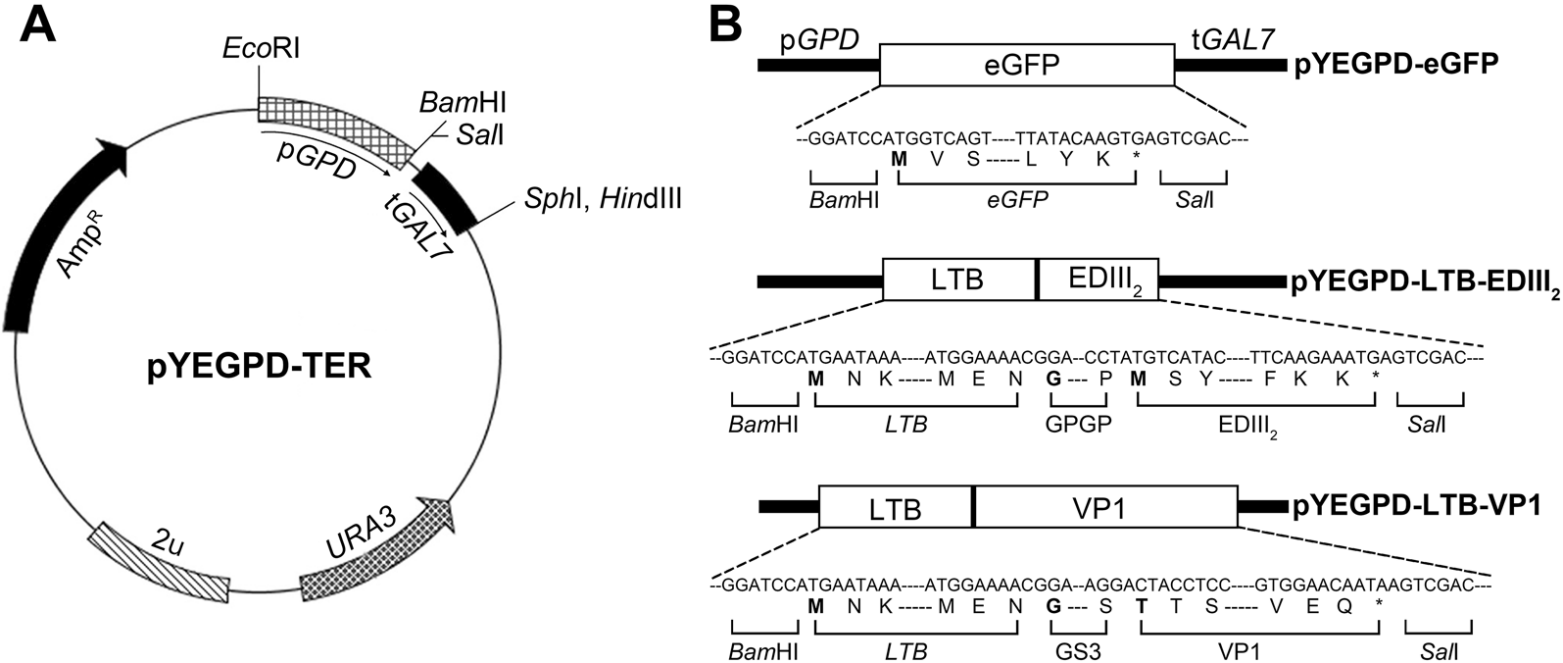
Schematic representations of expression vectors described in this study. All of the expression cassettes were cloned into the pYEGPD-TER (**A**) vector [13] using *Bam*HI and *Sal*I restriction enzymes

Yeast codon optimized eGFP (GenBank accession No: ON036474) was synthesized. *Bam*HI and *Sal*I restriction enzyme recognition sites were added before the start codon and after the stop codon, respectively. The resulting fusion gene was cloned in a pGEM T-easy vector for sequence verification. Subsequently the gene was released from the vector by *Bam*HI/*Sal*I and cloned into the pYEGPD-TER vector, which contained a constitutive GPD (glyceraldehyde-3-phosphate dehydrogenase) promoter and a Gal7 terminator (Fig. 9A). Using overlap-extension PCR, genes encoding EDIII_2_ and VP1 (GenBank accession No: KT452797.1 and ON036475, respectively) were cloned in-frame with the LTB encoding gene. GPGP (GlyProGlyPro) flexible linker [16] and GS3 (Gly_4_Ser-Gly_4_Ser-Gly_4_Ser) flexible linker [62] were inserted between these genes to allow for independent folding of EDIII_2_ and VP1, respectively. *Bam*HI and *Sal*I restriction enzyme recognition sites were then added before the start codon and after the stop codon, respectively. The resulting fusion genes were cloned using the pGEM T-easy vector for sequence verification. Subsequently, these genes were released from the vector by *Bam*HI/*Sal*I and cloned into the pYEGPD-TER vector (Fig. 9B). The recombinant pYEGPD-TER vectors were transformed into the *S. cerevisiae* 2805 strain using the lithium acetate method [63]. The primers used for each expression vector are listed in Additional file 3: Table S1.

### Northern blot analysis

After screening for positive transformants by colony PCR and *E. coli* back transformation [18], twelve random transformants were selected for expression analysis. The expression of these transformants at the transcription level was compared using Northern blot to identify those that were most expressed. The three transformants with the highest expression level were then selected for temporal expression analysis. To determine which transformants were most expressed, yeast cells were grown at 30 °C and harvested at day 3 post-inoculation. For temporal expression analysis, yeast cells were grown at either 30 °C or 20 °C and the cells in 40 ml YEPD were harvested at days 1, 3, and 5 post-inoculation, washed twice with dH_2_O, and then stored at − 80 °C for RNA extraction. Total RNA was extracted as described previously [64]. RNA samples were quantified using a spectrophotometer as 30 µg of each sample was placed on a 1.2% denatured agarose gel. The gels were then blotted onto Amersham Hybond™ membrane, crossed linked with UV, and hybridized in modified Church buffer. Detecting probes were prepared by isothermal (37 °C) amplification of the corresponding genes using random primers and cytosine labelled with ^32^P isotope.

### Real time RT-PCR

Quantitative real time RT-PCR was conducted as previously described to evaluate the expression levels of the target genes [65]. Each expression level was evaluated in triplicate for each transcript, with at least two independent preparations of the same RNA sample. Transcript levels relative to the amount of glyceraldehyde-3-phosphate dehydrogenase (GPD) of *S. cerevisiae* were used as an internal control. The primer pairs used for each target gene are listed in Additional file 3: Table S1. Statistical analyses were performed by *t*-test at *p* = 0.01 using SPSS software (IBM Corp., Armonk, NY). Different letters indicate significant differences between strains.

### Western blot analysis

Yeast protein was extracted consistent with methods previously described [18]. 100 or 200 µg of total soluble protein from each sample was mixed with 6 × SDS-PAGE loading buffer and either boiled (denatured) or not boiled (non-denatured) before being loaded onto 10% SDS-PAGE gel.

After gel separation, each gel was then blotted onto nitrocellulose membrane (Hybond™ Cytiva Life Sciences), and the target proteins were detected with specific primary antibodies (either rabbit anti-LTB or mouse anti-dengue envelope domain III) and AP-conjugated secondary antibodies. Western band intensities, which reflect the relative amount of target proteins in the samples, were determined using the Fiji ImageJ software package [66].

### GM1 ELISA analyses

GM1 ELISA was used to quantify the amount of pentameric assembled LTB fused antigens. In brief, 96-well microtiter plates were coated with 0.3 µg GM1 (Sigma G-7641) and incubated overnight. The next day, the plates were washed 3 times and blocked with 1% BSA for 1 h at 37 °C. LTB standard and yeast protein samples were added and serially two-fold diluted. The plates were incubated for 2 h at 37°C. After washing, the primary antibody (rabbit anti-LTB, 1:5000 dilution) was added and the plates were incubated for another 2 h at 37 °C. The secondary antibody was then added and the plates were left standing at 37 °C for an additional 2 h. Finally, after three washings, the plates were developed with AP substrate solution (prepared from AP substrate tablets—Sigma S0942-100TAB) and optical density was determined by their absorbance at 405 nm (Multiskan GO microplate reader ThermoFisher Scientific). Statistical analyses were performed by t-test at *p* = 0.01 using SPSS software (IBM Corp., Armonk, NY).

### FACS

Yeast cells expressing eGFP were harvested, washed twice with PBS, and subjected to FACS analysis using a BD FACS CANTOII flow cytometer with a 488 nm excitation laser line. The data was subsequently analyzed using FlowJo V10 software to determine average fluorescent intensities. Statistical analyses were performed by *t*-test at *p* = 0.01 using SPSS software (IBM Corp., Armonk, NY).

## Supplementary Information

**Additional file 1: Figure. S1 **Western blot analysis of temporal expression of LTB-EDIII2 from transformant #8 using anti-Dengue antibody. **A **LTB-EDIII2 expression was resolved under non-denaturing condition at 20 °C and 30 °C. **B **SDS-PAGE gel showed that an equal amount of protein was loaded on each lane. Lane 1: purified *E. coli*-expressed LTB as a positive control; Lane 2: a mock transformant cultured for 3 days as a negative control. Proteins were prepared from transformant #8, and cultured for 1, 3, 5 days after inoculation to the expression medium at 20 °C (lanes 3, 5, and 7, respectively) and 30 °C (lanes 4, 6, and 8, respectively).

**Additional file 2: Figure. S2 **Northern blot analysis (**A**) and Quantitative real time RT-PCR (qRT-PCR) analysis (**B**) of LTB-VP1 in a selected transformant (#4) under 20 °C and 30 °C conditions. **A **20 μg total RNA was loaded on each lane. RNA preparations from cells harvested at day 1, 3, and 5 days after cultivation at 20 °C (Lanes 2-4, respectively) and 30 °C (Lanes 5-7, respectively). Lane 1 contains RNA sample from a mock transformant cultured for 3 days at 30 °C as a negative control. GPD was used as an internal control and rRNAs are shown to indicate equal amount of RNA loaded on each lane. **B **qRT-PCR results of changes in expression of LTB-VP1 under 20 °C and 30 °C conditions are shown. No significant differences between 20 °C and 30 °C were observed.

**Additional file 3: Table S1. **List of Primers used in this study.

## Data Availability

All data for this study are included in this published article.

## Abbreviations

LTB: *Escherichia coli* Heat labile toxin B subunit
LTB-EDIII_2_: LTB fused to the envelope domain III (EDIII) of Dengue virus serotype 2
LTB-VP1: LTB fused to VP1 antigen of Foot-and-Mouth Disease Virus
LTB-scEDIII: LTB fused to the synthetic consensus sequence of envelope domain III of all four serotypes of Dengue virus
FMDV: Foot-and-Mouth Disease Virus
GPD: Glyceraldehyde-3-phosphate dehydrogenase
