# Supplementary material for: Improving expression and assembly of difficult-to-express heterologous proteins in Saccharomyces cerevisiae by culturing at a sub-physiological temperature
Source: Microb Cell Fact. 2023 Mar 23;22:55. doi: 10.1186/s12934-023-02065-7 (PMC10035479; doi:10.1186/s12934-023-02065-7)
Supplement: Supplementary file 1 — Additional file 1: Figure. S1 Western blot analysis of temporal expression of LTB-EDIII2 from transformant #8 using anti-Dengue antibody. A LTB-EDIII2 expression was resolved under non-denaturing condition at 20 °C and 30 °C. B SDS-PAGE gel showed that an equal amount of protein was loaded on each lane. Lane 1: purified E. coli-expressed LTB as a positive control; Lane 2: a mock transformant cultured for 3 days as a negative control. Proteins were prepared from transformant #8, and cultured for 1, 3, 5 days after inoculation to the expression medium at 20 °C (lanes 3, 5, and 7, respectively) and 30 °C (lanes 4, 6, and 8, respectively). [file 12934_2023_2065_MOESM1_ESM.pdf]

Additional file 1

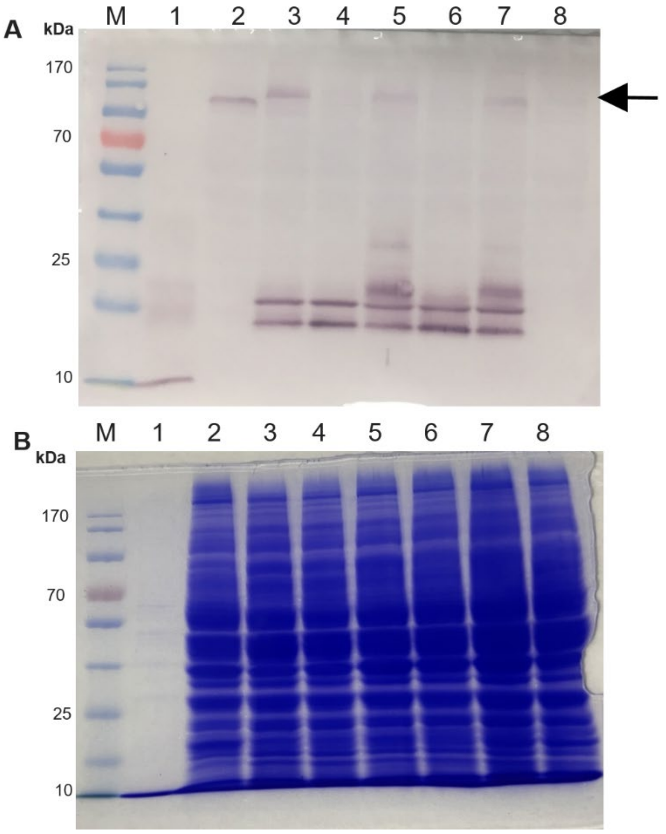

Fig. S1 Western blot analysis of temporal expression of LTB-EDIII$_2$ from transformant #8 using anti-Dengue antibody. **A** LTB-EDIII$_2$ expression was resolved under non-denaturing condition at 20 ºC and 30 ºC. **B** SDS-PAGE gel showed that an equal amount of protein was loaded on each lane. Lane 1: purified *E. coli*-expressed LTB as a positive control; Lane 2: a mock transformant cultured for 3 days as a negative control. Proteins were prepared from transformant #8, and cultured for 1, 3, 5 days after inoculation to the expression medium at 20 ºC (lanes 3, 5, and 7, respectively) and 30 ºC (lanes 4, 6, and 8, respectively).
