# Supplementary material for: Improving expression and assembly of difficult-to-express heterologous proteins in Saccharomyces cerevisiae by culturing at a sub-physiological temperature
Source: Microb Cell Fact. 2023 Mar 23;22:55. doi: 10.1186/s12934-023-02065-7 (PMC10035479; doi:10.1186/s12934-023-02065-7)
Supplement: Supplementary file 2 — Additional file 2: Figure. S2 Northern blot analysis (A) and Quantitative real time RT-PCR (qRT-PCR) analysis (B) of LTB-VP1 in a selected transformant (#4) under 20 °C and 30 °C conditions. A 20 μg total RNA was loaded on each lane. RNA preparations from cells harvested at day 1, 3, and 5 days after cultivation at 20 °C (Lanes 2-4, respectively) and 30 °C (Lanes 5-7, respectively). Lane 1 contains RNA sample from a mock transformant cultured for 3 days at 30 °C as a negative control. GPD was used as an internal control and rRNAs are shown to indicate equal amount of RNA loaded on each lane. B qRT-PCR results of changes in expression of LTB-VP1 under 20 °C and 30 °C conditions are shown. No significant differences between 20 °C and 30 °C were observed. [file 12934_2023_2065_MOESM2_ESM.pdf]

Additional file 2

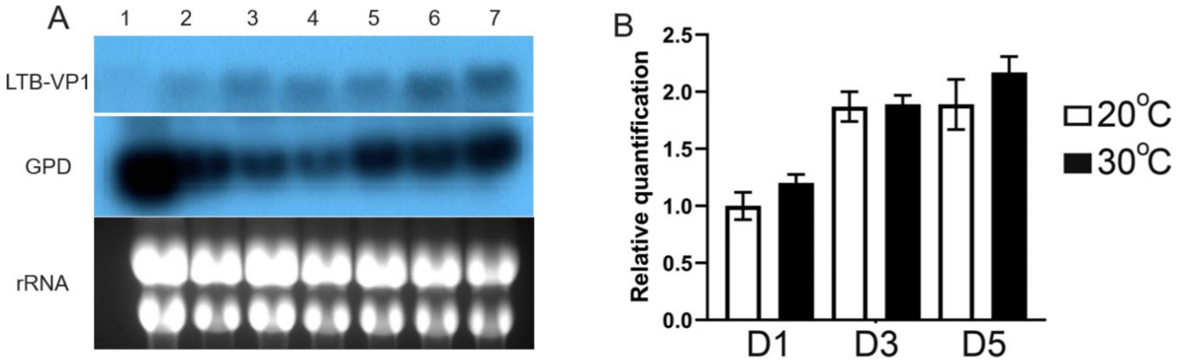

Fig. S2 Northern blot analysis (**A**) and Quantitative real time RT-PCR (qRT-PCR) analysis (**B**) of LTB-VP1 in a selected transformant (#4) under 20 ºC and 30 ºC conditions. **A** 20 μg total RNA was loaded on each lane. RNA preparations from cells harvested at day 1, 3, and 5 days after cultivation at 20 ºC (Lanes 2-4, respectively) and 30 ºC (Lanes 5-7, respectively). Lane 1 contains RNA sample from a mock transformant cultured for 3 days at 30 ºC as a negative control. GPD was used as an internal control and rRNAs are shown to indicate equal amount of RNA loaded on each lane. **B** qRT-PCR results of changes in expression of LTB-VP1 under 20 ºC and 30 ºC conditions are shown. No significant differences between 20 ºC and 30 ºC were observed.
