# Supplementary material for: Improving expression and assembly of difficult-to-express heterologous proteins in Saccharomyces cerevisiae by culturing at a sub-physiological temperature
Source: Microb Cell Fact. 2023 Mar 23;22:55. doi: 10.1186/s12934-023-02065-7 (PMC10035479; doi:10.1186/s12934-023-02065-7)
Supplement: Supplementary file 3 — Additional file 3: Table S1. List of Primers used in this study. [file 12934_2023_2065_MOESM3_ESM.pdf]

Additional file 3

Table S1. List of Primers used in this study

**Primer**	**Sequence (5' to 3')**
*BamH*I-eGFP-F	GGATCCGCATGGTCAGTAAGGGTGAAG
*Sal*I-eGFP-R	GTCGACTCACTTGTATAATTCGTCCA
*BamH*I-LTB-F	GGATCCGCATGAATAAAGTAAAATGT
GPGP-LTB-R	AGGACCTGGTCCGTTTTCCATACTGATTGCC
GPGP-EDIII$_2$-F	GGACCAGGTCCTATGTCATACTCTATGTGT
*Sal*I-EDIII$_2$-R	GTCGACTCATTTCTTGAACCAGTTGAGTTT
GS3-LTB-R	CACTTCCCCCTCCACCGCTGCCTCCCCCTCCGTTTTCCATACT
GS3-VP1-F	GTGGAGGGGGAAGTGGTGGAGGTGGGAGCACTACCTCCGCC
*Sal*I-VP1-R	GTCGACTTATTGTTCCACTGGTGCAACG
qRT-EDIII$_2$-F	ACATGTCTTAGGTCGCCTGATT
qRT-EDIII$_2$-R	GTCTCCGAATGGAGGTTCTG
qRT-GPD-F	GAGTTGCTATTAACGGTTTCGG
qRT-GPD-R	GGTCGTTCAAAGCAACAAC
qRT-VP1-F	CGGCGTTGGACAATACTACC
qRT-VP1-R	GCGGTGTGGTGCTGTATATG

- Underlines indicate recognizing sequences for corresponding restriction enzymes.
